# Pastoral Practices and Common Use of Pastureland: The Case of Karakul, North-Eastern Tajik Pamirs

**DOI:** 10.3390/ijerph15122725

**Published:** 2018-12-03

**Authors:** Teiji Watanabe, Shigeru Shirasaka

**Affiliations:** 1 Faculty of Environmental Earth Science, Hokkaido University, Sapporo, Hokkaido 060-0810, Japan; 2 Professor Emeritus, Tokyo Gakugei University, Koganei, Tokyo 184-8501, Japan; vanshira@rikkyo.ac.jp

**Keywords:** pastoralism, sustainability, commons, uneven pasture use, privatization of pastureland, trade-off, The Pamirs

## Abstract

This study describes pastoralism practiced in the Karakul village, Northeast of Tajikistan, and discusses its sustainability. Tajikistan introduced a market economy at independence in 1991, and pastoralism is now practiced on a family-unit basis. The families in Karakul graze livestock in their summer pastureland (*jailoo*) and move their livestock to winter pastureland around the village (*kyshtoo*). They make groups for pasturage with several families in *jailoo* and also in *kyshtoo*. Each group pastures their livestock every day, using a system called *novad*. In addition to *jailoo* and *kyshtoo*, they also practice pastoralism on two additional kinds of pastureland: *küzdöö* (spring pastureland) and *bäärlöö* (autumn pastureland). Still, now, the Karakul villagers use their pastureland as the commons: the Karakul village has not established private possession of pastureland even after a law enabled the division of common pastureland among individual families. Using the pastureland as the commons would be preferred by the local pastoralists. However, the free pasture access as the commons may result in a loss of sustainability as a trade-off. Regardless of privatization or the continued use of the commons, the possible development of the uneven use of the pastureland is inferred and should be avoided, and the introduction of a local management structure is urgently needed.

## 1. Introduction

Pastoral practices have always adapted to new and threatening challenges and found an outlet to cope with the constraints of mountain environments [1]. For example, a study explored the challenges that Afghan nomads and transhumant herders faced in maintaining their livelihood with emphasis on food insecurity and conflict, land access, and land appropriations [2]. A recent study [3] discussed transformations in the spatiality of semi-nomadic pastoralism and the impacts of assault rifles and mobile phones on spatiality in East Africa. Another study [4] compared the Inner Mongolia of China, with its declining quantity and quality of grassland, and the Republic of Mongolia, which had fewer severe problems, and found differences resulting from demographic changes. Population growth and the resultant establishment of commercial-oriented large-scale reindeer herding have resulted in serious social changes in the European tundra [5].

There are increasing publications on the transformation of pastoralism in Central Asia, including the Pamirs, in the post-Soviet era [6]. In the Fergana Range of Kyrgyzstan, degradation, growing pasture shortage, and resultant conflicts among pastoralists were examined [7,8]. In the eastern Alai Valley of the Kyrgyz Pamirs, the relationship between overgrazed pastureland and the grazing intensity was discussed [9,10]. Pastoralism above the cultivation limit was described in the same area [11,12], and diversified pastoralism as a survival and adaptive strategy was examined [13].

The Tajik Pamirs, which is characterized by a complicated aggregate topography with mountain ranges, basins, and gorges from 2000 m in altitude to more than 7500 m, occupies the eastern part of Tajikistan. The Tajik Pamirs administratively belongs to Gorno-Badahshan Autonomous Oblast (GBAO). There are some studies on pastoralism in GBAO (e.g., References [14,15]), with many of them investigating how pastoral practices have transformed. Another study [16] overviewed the post-Soviet transformation processes that are related to pastures as common property. The impact of land reform legislation that changed pasture tenure from a common resource to private property was discussed [17,18]. The ‘tragedy of the commons’ [19] or the ‘drama of the commons’ [20] has been discussed in some studies [17,21]. The ‘drama of the commons’ appears to be a ‘drama of responsibility’, where the vital interests of rural people and communities are at stake and grossly neglected by their own governments [1]. 

Transformation of pasture tenure from a common resource to private property also affects the sustainability of natural resources. In the wide area of the north-eastern and northern Tajik Pamirs [22], found overgrazing near a settlement, which constrains the sustainable use of natural resources. On the other hand, [23] found that some distant and hardly accessible summer pastureland show high livestock numbers. They concluded that unequal use of pastureland limits the sustainable use of natural resources. Productive development of the livestock sector will influence and determine future sustainability in the development of Tajikistan [24]. 

Pastoralism is a valuable survival and adaptive strategy [21]. How has pastoralism in north-eastern GBAO been transforming? The above pastoralism studies show that privatization is underway in GBAO; however, there are no studies on pastoralism in the north-eastern part of GBAO, i.e., the Karakul area. The Karakul area, located at the national border, neighboring with southern Kyrgyzstan, is one of the most remote areas in Tajikistan. Karakul and southern Kyrgyzstan have had strong interactions between their residents and economies because of their geographical locations. This legacy from the Soviet era is still observed. However, changes in the law, such as the prohibition to export livestock from Tajikistan and Kyrgyzstan, enforced in 2009, may cause additional challenges for the pastoralists in the area soon. In these regards, this study focused on the Karakul area.

This study has three objectives. The first objective is to describe the status of pastoralism in the Karakul village in north-eastern GBAO. Information on the changing status of pastoralism in the Tajik Pamirs is extremely limited and no information is available in the study area. Furthermore, the Karakul area is one of the remaining areas where no pastureland is privatized in the Tajik Pamirs despite the land reform legislation. Therefore, a detailed description of the current status of pastoralism in the study area, before the transformation begins, has a significant meaning. Additionally, the future transformation from the common use to private use may influence the sustainability of the pastureland. The second objective is to discuss the relationship between these two modes of pastureland use for the sustainability of the pastoral community. The third objective is to briefly discuss the connectivity of the current pastoral marketing with the neighboring country of Kyrgyzstan, which will be an important basis for future policy on sustainability in the area. 

## 2. Materials and Methods 

### 2.1. Study Area

**Figure 1:**
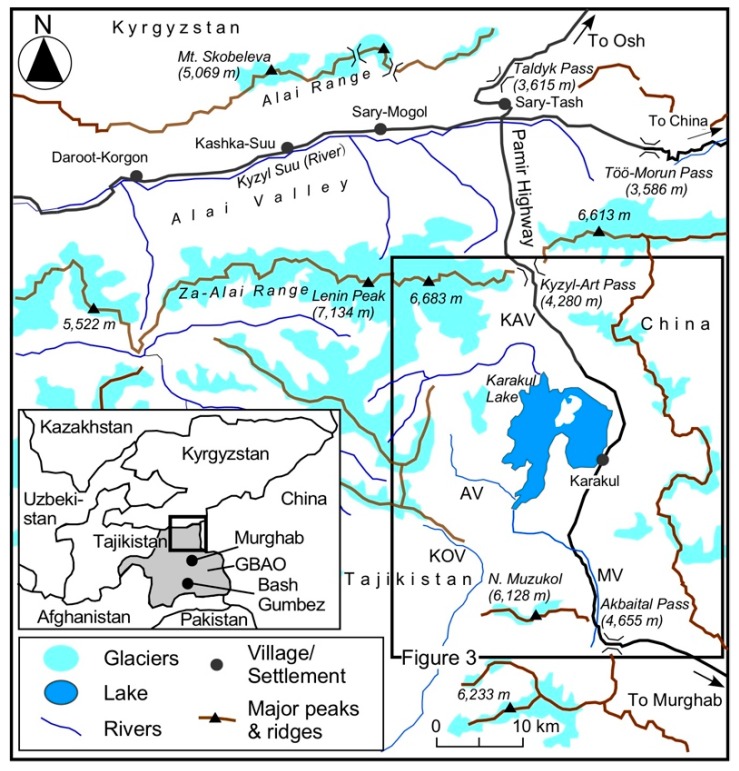
The study area. KAV: Kyzyl-Art Valley; AV: Akjilga Valley; KOV: Kokuibel Valley; MV: Muzukol Valley.

The area of GBAO is approximately 64,000 km^2^ and its total population is estimated at 216,000 [25]. The Murghab district (*rayon*) has an area of 38,300 km^2^ and is located in the eastern part of GBAO, where approximately 16,000 people reside [25]. The Karakul village is situated on the east side of the Karakul Lake in north-eastern GABO and it is the only settlement in the study area (Figure 1). The village mayor and officers said that the population and family numbers of the Karakul village, which in 2010 were 804 and 163, respectively, and almost all of them were Kirghiz people, who came to this area with sheep, goats, and yaks from the north and the east. Only one family was Tadzhik. They also estimated that approximately 130 families (80% of inhabitants) rely mainly on livestock farming, and about 20% are public employees (the staff of the village office and school teachers). The most prosperous family kept 300 yaks in addition to approximately 1000 sheep/goats and employed two shepherds (*chaban* is the local name) as of August 2014.

The climate of the study area is characterized by extreme aridity and low temperatures. The annual mean precipitation and the annual mean air temperature in the Karakul village are 80.6 mm and −3.6 °C, respectively (compiled data from Reference [26]). The local residents informed us that severe winters with extremely low temperatures strike the area approximately once or twice a decade, causing the death of a large quantity of livestock. In addition, heavy snow greatly affects yaks because they graze outdoors and cannot find dry grass underneath the thick snow in winter. 

The land use and land cover in the study area (southern part of the Karakul Lake, Akjilga Valley, and northern part of the Kokuibel Valley) can be classified into five categories which are composed of bare ground (about 67%), steppe and alpine vegetation (13%), water (12%), snow and ice (7%), and others (1%) [27]. Farming is not practical because of low temperatures (the monthly mean air temperature exceeding 10 °C occurred only in July and August in the Karakul village by our measurements, the method of which will be outlined in the next section) and a lack of irrigation water. The residents in the Karakul village can obtain only a small amount of grass for the winter to the north of the village.

### 2.2. Data Collection and Methods

To prepare the distribution map (of the locations and names) of summer and winter pasturelands, field observations were conducted. We visited all *jailoo* in the Akjilga Valley, and most in Kokuibel and Muzukol valleys. However, we did not visit the *jailoo* in Kyzyl-Art Valley, but observed them from a vehicle. Regarding *kyshtoo*, we visited only some, as many of them are in the buffer zone between the national border of China and the Pamir Highway, to which no access by foreigners is allowed. The locations of *jailoo* and *kyshtoo* that we were not able to visit were confirmed in interviews with the village mayor, officers, and pastoralists. 

To understand the current pastoral practice, we conducted pilot surveys in the Karakul village and the Akjilga Valley during 2003 and 2007 and extensive fieldwork mainly in the Akjilga, Kokuibel, and Muzukol valleys during 2010 and 2015, totaling 53 days. We focused on topic-specific interviews with local pastoralists, which include the number and type of livestock, seasonal migration patterns, and the number and duration of work for employed shepherds, if they employ. The total number of the interviewed pastoralists was 50: all eight families in Jalang Jailoo and 1–4 families in each of the other *jailoo* that were visited. We also met the village mayor, vice-mayor, and officers to conduct general interviews including population, major occupations of the villagers, the total number of livestock in the area, and infrastructure. In many cases, local guides interpreted our interviews when the informants could not command English. In addition, statistical data were collected to examine the livestock number in GBAO and the Murgab district. 

Climate datum was available only for the Karakul village. To compare the air temperatures and precipitations among three different altitudes, we installed flowing sensors and data loggers in the Karakul village (39°00’39” N, 73°33’34” E, 3930 m a.s.l.), Jalang Jailoo (38°46’37” N, 73°06’11” E, 4094 m a.s.l.), and Sai-Konush Jailoo (39°00’27” N, 73°06’18” E, 4348 m a.s.l.) in August 2014. The equipment included thermistor sensors connected to TR-52 data loggers (T&D Corporation, Matsumoto, Japan) to measure air temperatures at 1-hour intervals, a Rain Collector II (Davis Data Instruments, Hayward, CA., USA) connected to the HOBO Pendant Data Loggers (Onset Computer Corporation, Bourne, MA., USA) to measure precipitation, and a Soil Moisture Smart Sensor S-SMA-M005 (Onset Computer Corporation, Bourne, MA., USA) connected to the HOBO Micro Station data loggers (Onset Computer Corporation, Bourne, MA., USA) to measure soil moisture at a 10-cm depth from the ground surface at 3-hour intervals. The precipitation was not measured during the snow period, and the average soil moistures were calculated only during the period of vegetation growth (May to August). Table 1 summarises the measured climatological data at the three sites. The measured year was much warmer than usual (the annual mean temperature in the Karakul village is −3.6 °C, as already mentioned). More precipitation is likely to occur in the six months from November to March because the seven-month precipitation was 26.2 mm and the annual mean precipitation is 80.6 mm [26]. A very approximate estimate of the annual precipitation in Sai-Konush would attain 225 mm. 

## 3. Pastoralism in the Post-Soviet Era 

### 3.1. The Private Possession of Livestock after the Collapse of the Soviet Union

**Figure 2:**
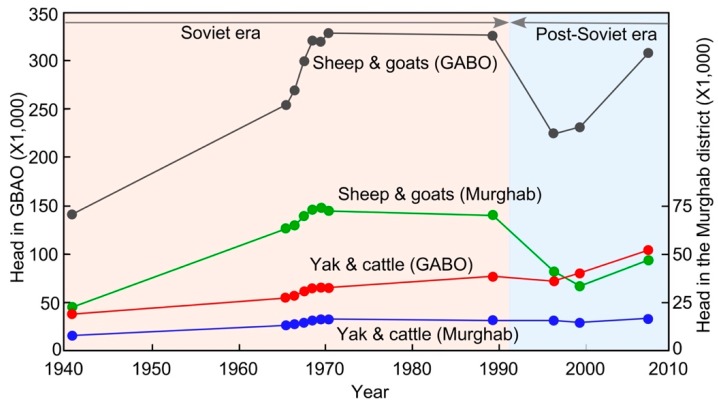
The changes in livestock number in GBAO and the Murghab district from 1941 to 2008 (Data source: GBAO Statistic Committee and Stat Passport of Rayons, 2009 and Ob1Stat GABO).

Figure 2 shows the change in the quantity of the livestock in GBAO from 1941 to 2008. Feed was introduced, imported, and distributed to the *kolkhoz* (collective farms) in the Tajik Pamirs during the Soviet era. Additionally, veterinarian services were substantial, which contributed to the rapid increase in the number of livestock from the 1940s to the end of the Soviet era. Then, *sovkhoz* (state-owned farms) were dismantled in 1998, and livestock, which *sovkhoz* had owned, were distributed among local families. All pastoral families in the area became *fermer* (personal management farmers). The basic policies of the distribution in Karakul were as follows: (1) the period served as laborers in the *kolkhoz/sovkhoz*, and (2) the number of family members. As a base, five sheep/goats (three females and two males) and two yaks were distributed per inhabitant who did domestic animal-related work in *sovkhoz*. Some families who received few animals sold their livestock and emigrated from the village. The governmental provision of kerosene, coal and other necessities stopped after independence in 1991. The collapse of the Soviet Union caused food and fuel shortages as seen in other regions of former Soviet countries. 

The law relating to *fermers* in Tajikistan was amended in 2005, and the private management of livestock and agriculture were legalized as so-called *legalizatsiya*. There was to be only *fermer* management in Karakul, and all *fermers* were required to join the Association of *Fermers* (the organization of farmers and pastoralists for the whole country). In Karakul village, the pastureland and grassland have been national land and the inhabitants were only given usufruct of the pastureland. The national pastureland of the Karakul village is currently supposed to be managed by the village authority in cooperation with the local Association of *Fermers*.

### 3.2. Livestock and the Joint Pasturing

Today, yaks, cows (milk cows), sheep, goats, and donkeys are kept as livestock in the Karakul area. Horses and camels had been bred as an important means of transportation in the *sovkhoz* era, but they are not kept anymore. Various statistics and studies suggested that by 2004, the number of livestock in Tajikistan had recovered to 1991 levels [17]. The number of livestock in GBAO and the Murghab district show a similar trend (Figure 2). 

Our interview with the vice-mayor of the village in August 2013 suggested that approximately 14,000 sheep/goats were grazing in Karakul at the end of the *sovkhoz* (Soviet) era, which would have been about 20% of the total output in the Murghab district (69,327 heads) in 1991 (Figure 2). After independence in 1991, the number greatly decreased in the Murghab district (Figure 2). Poor inhabitants sold much of the distributed livestock, tending to keep the large livestock (yaks) if possible but selling their sheep/goats, which coincides with the observed trend in the Murghab district. An interview with the vice-mayor of the village in 2013 indicated that the estimated number of livestock in the Karakul area were 10,000 sheep/goats, 100 cows, 2500 to 3000 yaks, and 90 donkeys, and that there is an increasing trend of the livestock number in the area. 

### 3.3. Joint Pasturing: Novad

In parts of the Pamirs, rotational joint pasturage is practiced by some families who own small numbers of livestock. Such a system is called *kezu* in Kyrgyzstan [18], *kezüü* in the eastern Alai Valley and *novad* in the western Alai Valley of southern Kyrgyzstan [13]. The people of Karakul call it *novad*, meaning ‘we do in turn’. They make *novad* for sheep/goats and grownup yaks in the *jailoo* (summer pastureland) in summer and for sheep/goats in winter. Only one shepherd takes care of *novad* in Karakul. 

**Figure 3:**
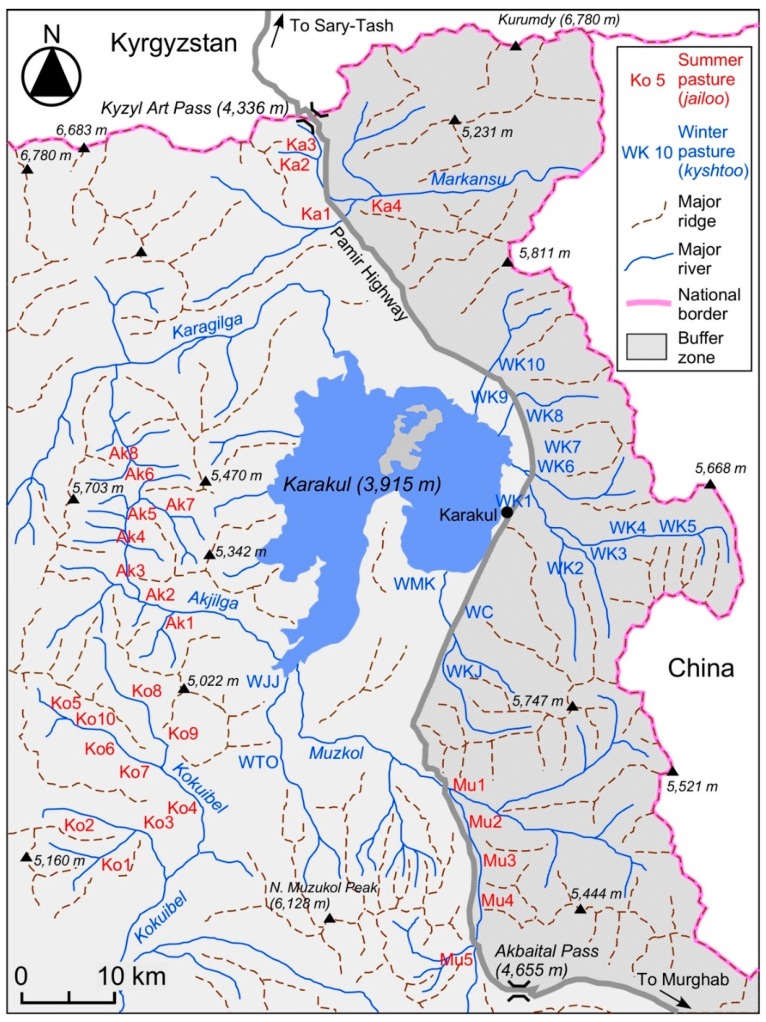
The locations of summer and winter pastures in Karakul (2015) and the buffer zone between the national border of China and the Pamir Highway. Summer pasture (*jailoo*) = Ka1: Eshan-Rabat; Ka2: Bulak; Ka3: Kyzyl-Art; Ka4: Ui-Bulak; Ak1: Kosh-Jilga; Ak2: Chat; Ak3: Zulumart; Ak4: Istik; Ak5: Sai-Konush; Ak6: Bel; Ak7: Maamat; Ak8: Kara-Chim; Ko1: Apak; Ko2: Kara Chim; Ko3: Kök-Dün; Ko4: Dangi; Ko5: Tash-Jilga; Ko6: Jalang; Ko7: Tuura-Bulak; Ko8: Kül-Airik; Ko9: Arka-Jalang; Ko10: Onoi Gösh; Mu1: Kyzyl-Jeek; Mu2: Muzkol; Mu3: Shor-Bulak; Mu4: Don-Shiber; Mu5: Chini-Suu. Winter pasture (*kyshtoo*) = WJJ: Jangi-Jer; WTO: Tumushuk-Ötök; WMK: Döö-Ötök; WC: Chöbök; WKJ: Kara Jilga; WK1: Karakul; WK2: Karteke; WK3: Kara-Art 1; WK4: Kara-Art 2; WK5: Kara-Art 3; WK6: Kara-Art-Agin; WK7: Köyöndu; WK8: Kum-Chukur; WK9: Kara-Shilarjin; WK10: Kök-Chukur.

In summer, families using the same *jailoo* gather and make *novad* for their yaks and sheep/goats. Generally, five to six families make one *novad* with 200 to 500 sheep/goats in Karakul. As a general rule, one person from each family acts as a shepherd on a rotational basis every day. The people of Karakul make 12 to 13 *novad* in the village for their sheep/goats every winter. For example, seven families, who used Jalang Jailoo (Ko6 in Figure 3) in 2011, made two *novad* for 46 yaks and 457 sheep/goats, for which two shepherds were necessary. The owners pay the shepherds a fee for every 50 sheep/goats; for example, if family M owns 150 sheep/goats, the family would pay three units of the fee.

The number of the winter *novad* for sheep/goats in Karakul varies from year to year. There were about 15 winter *novad* in 2013/14 and 11 in 2014/15. They do not name each *novad* in Karakul, unlike in the Alai Valley [13]: sometimes they call the *novad* by the organizer’s name. Families using the same *jailoo* in summer may not belong to the same *novad* in winter. 

### 3.4. Locations of Seasonal Pastureland

Since the nomadic era, pastoralists have had four kinds of pastureland to stay in each season. The Kirghiz people call their areas of residence and pastureland (*jaiyt* as a general name) different seasonal names: *kyshtoo* for winter/spring, *bäärlöö* for spring, *jailoo* for summer, and *küzdöö* for autumn. 

Figure 3 shows the location of *kyshtoo* (W; 15 places) in the winter season and *jailoo* (four groups of Ka, Ak, Ko, and Mu; in total 27 places) in the summer season. The distributions of *bäärlöö* and *küzdöö* are excluded in Figure 3 to avoid complications. Our interviews with the inhabitants show that each *kyshtoo*, *jailoo*, *bäärlöö* and *küzdöö* has a name. *Bäärlöö* and *küzdöö* are located at least 4 to 5 km away from the Karakul village. The far-off *bäärlöö* and *küzdöö* are 10 to 20 km away. In addition, *bäärlöö* and *küzdöö* also exist in the vast buffer zone between Tajikistan and China (the Tajik side is fenced with barbed wire along the Pamir Highway; see Figure 3). Recently, people have built huts made of sun-dried bricks in their *bäärlöö* and *küzdöö*.

#### 3.4.1. *Kyshtoo* and Pasturage in Winter

**Figure 4:**
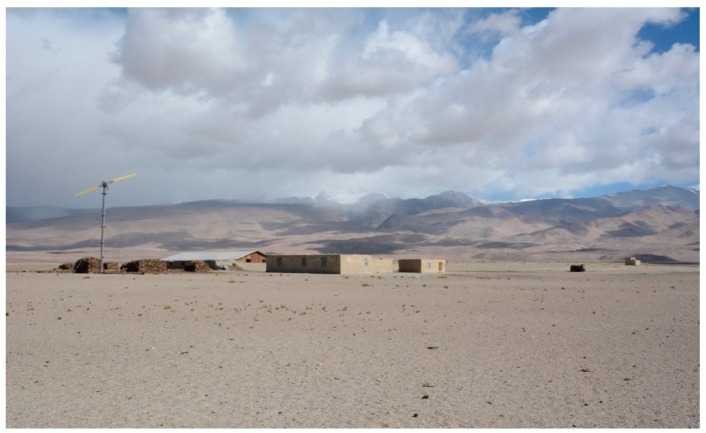
An isolated *kyshtoo* (Photograph: S.S., September 2015).

The Karakul village is a *kyshtoo* and there are 16 *kashars*, i.e. sheds of livestock in winter, remotely located in the *kyshtoo* (Figure 4). *Kashars* are also located in *bäärlöö* and *küzdöö*, which are located in the vast buffer zone between China and Tajikistan. There are sheds called *koloo* that can accommodate 200 to 300 sheep/goats. In each *kashar*, there is also a hut with a partial roof for baby sheep/goats, called *kozu-kapa*, in addition to a house for the shepherd’s family. 

In the winter season, the families that own more than 100 sheep/goats often use their *kashar* located outside the Karakul village. Families with fewer than 100 sheep/goats usually live in the Karakul village during the winter season. In addition, there are about ten families who breed sheep/goats only by themselves in Karakul during the winter season. Such families make their livestock *novad* in the pasture around the village.

Livestock owners now entrust their sheep/goats and yaks to shepherds in winter, which started in about 2010. There are at least 25 shepherds, who are all local people, to undertake the pasturing in winter. Some shepherds exclusively work for livestock. The others work part-time and are called *sabashka* in Russian. They take construction-related work in summer and work as shepherds only in autumn and winter.

Members of the *novad* receive the sheep/goats from the shepherd in the evening, and each family fixes the hair of all sheep/goats and cleans their bodies. The sheep/goats that come back from pasturage nurse their babies in spring. When babies are born in the *kashar*, the owners have to pay 3 Som per head to the shepherds in addition to the trust fee. Babies are handed to the owners immediately, and the babies are provided with hay.

The local people do not keep their yaks in their huts, even during winter. Male and female yaks are bred together outdoors. However, males live apart from females and babies in winter. The male group joins the female group during the time of mating from April to September. The owners and the employed shepherds inspect their group once a week to observe the breeding situation of their yaks. Some families trust the management of yaks to shepherds from October to April. 

Cows stay in *kashar* in the *kyshtoo* and in the Karakul village. The cows are taken from the *kyshtoo* to the pastureland, which is 5 to 6 km away, in the morning, and they are brought back to the *kyshtoo* in the evening. Milk cows are bred in sheds in the *kyshtoo* in winter and in *jailoo* in summer. The owners do not entrust the breeding to shepherds.

#### 3.4.2. *Jailoo* and Pasturing in Summer

**Figure 5:**
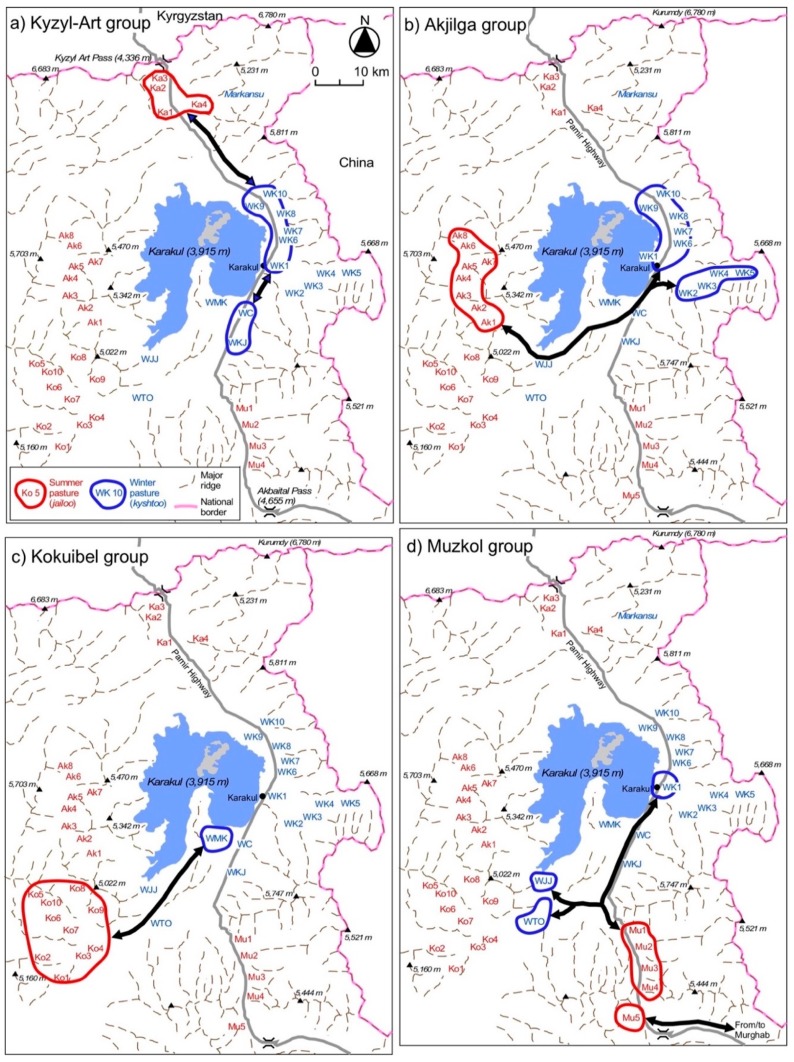
The major movement patterns between *jailoo* (summer pastureland) and *kyshtoo* (winter pastureland).

**Figure 6:**
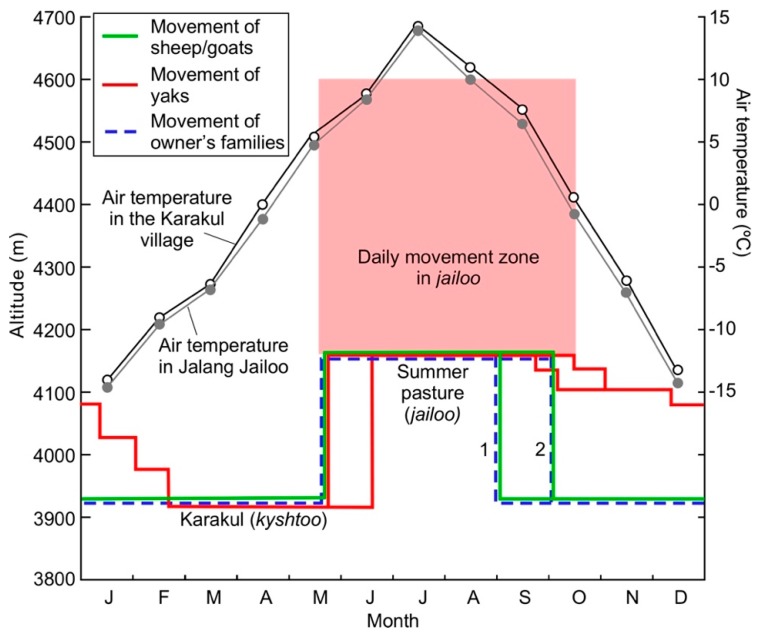
The monthly mean air temperatures in the Karakul village and Jalang Jailoo (Ko6), and the mobility of livestock and pastoral farmers who migrate between the Karakul village and Jalang Jailoo. Depending on the locations of the *jailoo*, the altitudes where the livestock move every day greatly vary. In the case of Jalang Jailoo, they vary from 4094 m to about 4600 m. The sheep/goats staying in Jalang Jailoo in summer do not stay in *bäärlöö* in springtime, meaning that they go directly from the village to the *jailoo* in one day. They stay in *küzdöö* for only one night in autumn when they go down to the village. Yaks move from the village to the *jailoo* by taking a few days, but from the *jailoo* to the village taking 4–5 months using *küzdöö* in autumn/early winter time.

A trip between the Karakul village and their *jailoo* (Figure 5 and Figure 6) takes one to two days on foot. Karakul people use *jailoo* in the Kyzyl-Art Valley (Ka in Figure 3 and Figure 5), the Akjilga Valley (Ak), the Kokuibel Valley (Ko), and the Muzkol-Akbaital Valley (Mu). The number of *jailoo* used in the Karakul area in 2010 was as follows: 4 in the Kyzyl-Art Valley (Ka1-4), 9 in the Akjilga Valley (Ak1-8), 10 *jailoo* in the Kokuibel Valley (Ko1-10), and 5 in the Muzkol-Akbaital Valley (Mu1-5). However, the interviews conducted in 2012 showed that five *jailoo* in Ka1-4 and Mu1 were not used, indicating some annual fluctuations. 

One *jailoo* is most frequently used by three to five families, though sometimes only one family will use a *jailoo*. The most heavily used *jailoo* was Jalang Jailoo (Ko6), with eight families in August 2015. Fifteen families in total who use *jailoo* in the Kokuibel Valley grazed their 300 female yaks in 2015. One shepherd cared for these 300 yaks when the yaks descended from the *jailoo*. 

The owner families who use the Akjilga Valley (Ak1–8) gather most of the yaks in Kara-Chim Jailoo (Ak8) every autumn. The total number of yaks owned by the 15 to 20 families is approximately 800. One person from the families in the Kara-Chim Jailoo becomes a shepherd. The shepherd is paid 60 Som/head/month for yaks and 20 Som/head/month for sheep/goats, as of 2016. In addition, one person from the families helps the shepherd on a weekly rotational basis. The shepherd and the supporting person watch over the yaks in the *jailoo* until December. The two people return to Karakul afterward, but the yaks remain in Ak8.

Shepherds are men without exception among the Kirghiz. Women milk the cows with their husbands or children in the morning and evening and do housework while the animals are taken to the pasture. The most important work of the women is making butter and cheese by using a separator, which was brought in after World War II. 

Calves of yaks are taken to the pasture around the families’ houses in the *jailoo* where the family members can see them. Because milk cannot be collected without the calves of yaks, people always watch where baby yaks are grazing.

A successor usually uses the *jailoo* that his parents used, though some families will change their *jailoo*. For example, family MK changed from Onoi Gösh Jailoo (Ko10) to Jalang Jailoo (Ko6) in 2014. The family changed their *jailoo* because Onoi Gösh Jailoo was too far from Karakul and because the cost of gasoline increased. In addition, their parents died. People usually change their *jailoo* when someone in the immediate family or a relative dies. 

Some families make one group for the movement of their livestock, and some of those families may act as shepherds. Most people move to the *jailoo* with their children and pensioners in summer. Therefore, the Karakul village becomes ‘empty’ in summer. The village officers told us that there are no limitations about the use of *jailoo* in the village of Karakul. Therefore, villagers can freely choose to use any *jailoo* in the village. 

### 3.5. Seasonal Movement of Livestock

Figure 5 shows the horizontal movement patterns of the major livestock groups between summer and winter pasturelands. The families of these four groups are residents of the Karakul village. One exception is the users of the Chini-Suu Jailoo (Mu5); this family is from Murghab (Figure 1). Figure 5 demonstrates that many families migrate their livestock between the summer *jailoo* and the winter *kyshtoo* without staying in the Karakul village (Wk1). Families with especially large numbers of livestock do not stay in the village, even in winter. This is one of the major characteristics of pastoralism in the area. 

Figure 6 is a schematic diagram exemplifying the vertical mobility of livestock and pastoral farmers, who migrate between the Karakul village and Jalang Jailoo (Ko6). Eight families use Jalang Jailoo as mentioned above. They usually use a truck to transport the family members and sheep/goats from the village to the *jailoo*, though the yaks always walk. They graze their livestock on the grass-covered alluvial fan formed by a tributary river called Jalang Suu, which flows from the south. Most of the *jailoo* in the study area are located on the alluvial fans. The grass on the fans is sustained because meltwater from small glaciers in the uppermost area of the tributary valleys is supplied to the fans. The soil on the fans in the *jailoo* has a high water content during the summertime (Table 1). Thus, the distribution of the *jailoo* in the study area is strongly related to the availability of the stream water delivered from the small glaciers in the tributary valleys. 

The families and sheep/goats staying in Jalang Jailoo go down to the Karakul village in two days with a one-night stay in *küzdöö*. Yaks, on the other hand, stay in several *küzdöö* on the way down to the village (Figure 6). 

#### 3.5.1. Movement from *kyshtoo* to *jailoo*

The people of Karakul start to put their livestock out to *jailoo* in the middle of May and are finished around June 20 (Figure 6). Some families arrive at their *jailoo* in the middle of June because their children take their final examinations in school in early June. The altitudes of the *jailoo* vary greatly: for example, Dangi Jailoo (Ko4) is 3933 m (the lowest *jailoo*), Jalang Jailoo (Ko6) is 4094 m, and Maamat Jailoo (Ak7) is 4442 m (the highest *jailoo*). They hire a truck to transport their belongings for daily life, which takes approximately two hours from the Karakul village. They may carry females and babies of their sheep/goats by truck later. 

Yaks, including babies, may go to *jailoo* by themselves without the guidance of shepherds. This also happens when they leave their *jailoo* for the *kyshtoo*. The owners believe that this happens because of the routine round trip between their *kyshtoo* and *jailoo* that they have taken every year since the age of *torpok* (common name of a yak baby). When deep snow arrives, yaks may spontaneously come back to their *kyshtoo*, even if the owner leaves them in their *jailoo*. Therefore, the local people often say that yaks remember the way back.

#### 3.5.2. Movement from *jailoo* to *kyshtoo*

The time to move from *jailoo* to *kyshtoo* is decided by the snow depth. When the snow depth exceeds about 5 cm at the end of September or at the beginning of October, sheep/goats leave their *jailoo* (Figure 6). When there is less snow, they may leave their *jailoo* in December or even in January, which may happen once a decade.

Because the school term begins in September, some families with school children leave their livestock with a family member, and the rest of the family members may return to Karakul at the end of August (the broken line 1 in Figure 6). Otherwise, all family members with children return to Karakul, and then only husbands come back to the *jailoo.*

Sheep/goats are the first to go down from *jailoo* to *kyshtoo*, followed by yaks. Sheep/goats can move and eat dry grass if the snow depth is less than approximately 5 cm. They gradually descend the mountain pastures with shepherds (Figure 6). They leave the *jailoo* in the morning and stay in their *küzdöö* around the lake until the next day before they arrive at *kyshtoo*.

Although yaks are extremely resistant to the cold, they brush snow on the dry grass off with their mouths, not with their forefeet. Therefore, they can eat dry grass when the snow depth is less than 20 cm. The yaks in Kara-Chim Jailoo (Ak8) gradually return to Karakul (Wk1) via Jangi-Jer (WJJ) with no shepherds when the snow depth is approximately 20 cm (usually the end of December or the beginning of January). Local people say that ‘yaks have a map’ because of this. If the snow depth is less than 20 cm, the yaks will winter in the *jailoo* (Ak8). The winter of the 2013/14 was such a case, but this only happens once or twice a decade. 

## 4. Discussion

### 4.1. Privatization or Common Use of Pastureland? 

As the foregoing section described, the pastoralism practiced in the Karakul area has essentially remained unchanged since the end of the Soviet era. One of the most important concerns related to pastoralism in the Tajik Pamirs is the privatization of pastureland, which is now possible [16,18,28]. The Tajik government and development agencies changed from an open-access regime to a private property regime, which succeeded only in some areas of the country. A study [17] by quoting Reference [29] mentioned that the privatization of the pastureland for winter (*kyshtoo*) and autumn (*küzdöö*) occurs in different stages. The privatization of pastureland is likely to increase in the future. However, there are villages which have not privatized their pastureland, such as the Karakul area. 

The regime used to manage land with natural resources affecting both the environmental sustainability of the natural resources and accessibility by the users. There are four kinds of agreements on access to natural resources by users [30]. (1) Open-access regime: this regime does not control the members nor are the resource users identified; (2) common property or group property regime: resources are held by a limited group as common property and their use is established through an internal rule; (3) private property or individual property regime: natural resources are owned and are managed as personal property by an individual or a family; and (4) state ownership or government property regime: natural resources are owned by the nation, and the nation enforces access for users. Extensive observations were conducted in the Pamirs [17]: these authors did not observe any cases of category (1) above, but they found cases of the remaining three types in the eastern Tajik Pamirs. However, categories (1) and (4) are most likely the same, de facto, in the study area because of the lack of governmental control. Our field surveys found that the pastureland in the Karakul area is best categorized as category (2) above.

Our interviews conducted in 2015 showed that the Bash Gumbez village (Figure 1), with approximately 70 families in southern GBAO, made their pastureland private possessions in 2005. Pastureland was distributed to families, although some families in the village still used joint pastoralism. Our interviews in the surrounding villages in the same year suggested that the Bash Gumbez village was the only village in the area that had privatized its pastureland. Therefore, the Karakul area is not the only exception where pastoralists have not privatized pastureland, as privatization has been reported elsewhere [17].

Although livestock became personal possessions after independence, livestock owners have recognized the advantages of common pastureland. Our interviews also suggest that the residents of such villages understand the importance of joint usership of pastureland for sustainable pastoralism.

**Figure 7:**
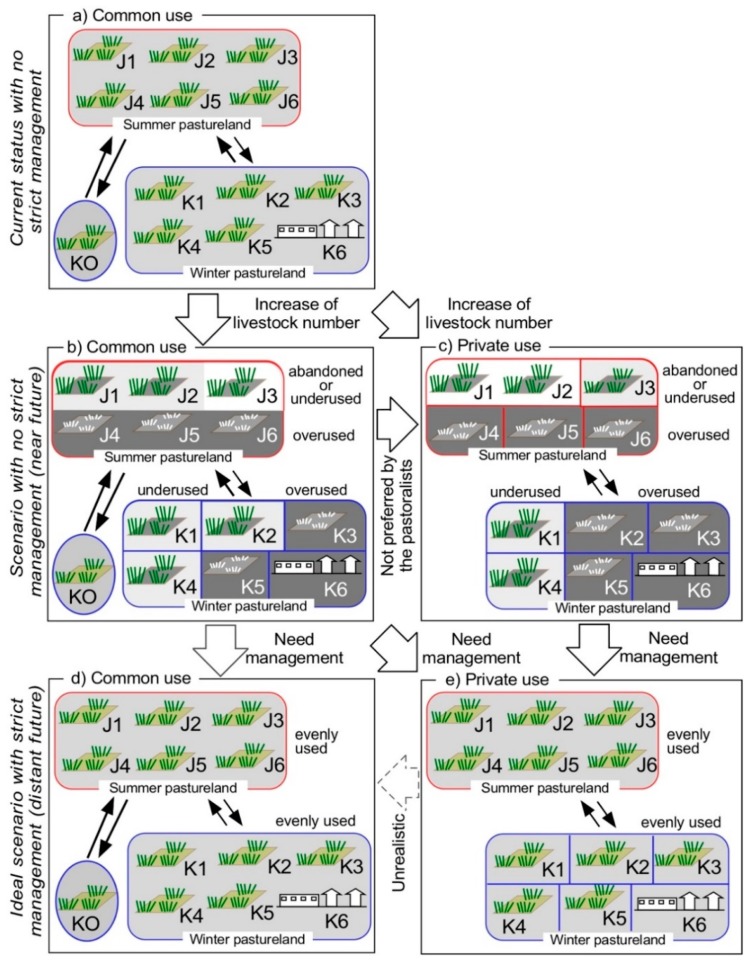
The current pastureland tenure and its future scenarios in the Karakul area. J1-6: *jailoo*; K1-6: *kyshtoo*; KO: Outsiders. a: current common-use status with no strict management; b: scenario of near-future status with no strict management (common use); c: scenario of near-future status with no strict management (private use); d: scenario of distant-future status with strict management (common use); and e: scenario of distant-future status with strict management (private use).

To obtain the right to use national land, this requires negotiation with the district Land Committee, which became possible by the law ‘On *Dekhan* Farms’ about personal farm (pastureland) management [17]. Although livestock owners must pay a user tax of 0.70 USD/ha for pastureland in some districts of GBAO, the pastureland tax system is not applied to the Murghab district [17] because of the difficult natural environment. In the case of the Karakul area, the privatization of the pastureland is not observed by now, as shown in Figure 7a. 

### 4.2. Pastureland Management: From Current Status to Future Possibilities 

The pasturelands are currently used in accordance with an unwritten code among the pastoral users in the Karakul area. This has been effective until now, indicating that the local pastoralists would not benefit from privatization. Many of them have not even recognized the law ‘On *Dekan* Farms’ because the Karakul area is one of the most remote areas from the capital city of Dushanbe and the enforcement of new laws/policies takes a long time to spread. Similar situations often happen in other issues including the illegal consumption of wildlife resources in the study area under the law of prohibition of wildlife hunting [31]. In the distant future, therefore, privatization will gradually proceed in the area, even though the local pastoralists would not prefer this.

The interviewees in the Karakul area observed the lack of a proper management system, which is supposed to be practiced by the village authority with help from the local Association of *Fermers,* as mentioned earlier. The interviewees also highlighted the lack of knowledge that the village authority possessed on pastureland usage. This means that the management structure exists but needs to function within the proper system. Therefore, the Karakul area needs a strong leadership to function the management of pastureland by establishing a pasture committee. Furthermore, competition among pastoralists has increased elsewhere in the eastern Tajik Pamirs because of the increase in the livestock number [22]. The continuous increase in the number of livestock especially among the prosperous owners in the Karakul area will require the adequate management of pastureland use.

**Figure 8:**
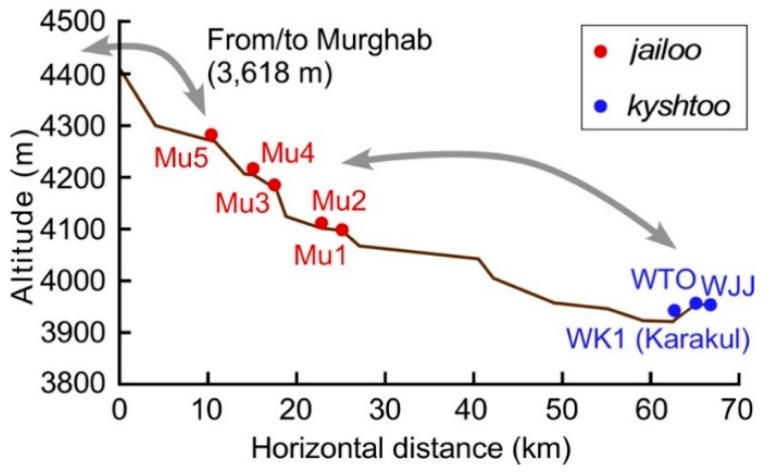
The longitudinal profile of the seasonal pastoral movement of the Muzkol group. Note that *jailoo* Mu5 is used by the outsiders from Murghab (3618 m).

The current pastoral practices with no strict management (Figure 7a) will soon shift to either of the scenarios shown in Figure 7b,c. If the current status with no strict management continues, the pastureland would degrade through development and overuse in the easily accessible *jailoo* and *kyshtoo*, as well as abandonment or underuse in the remote pastureland (Figure 7b). In the Soviet era, the state government supplied trucks that were necessary for livestock migration. Now, it is challenging to access pastureland distant from villages [32,33]. As described in the example of Family MK, successors often use different pasturelands because of the difficulty in access and because of the large costs of seasonal livestock migration, which also contributes to the uneven use. Many village authorities in GBAO now allow outsiders to access pastureland belonging to villages. The pastureland of Karakul is not an exception; de jure, it is available to outside villagers. One family came from Murghab to Chini-Suu Jailoo (Mu5 in Figure 5) in May and stayed there until the end of October (Figure 8). This outside family from Murghab brought 200 yaks to the pastureland in Chini-Suu Jailoo. They started to use the *jailoo* in 2010 because the pastureland in Murghab was not sufficient for their needs. Although outsiders have not been observed in other *jailoo* in Karakul, the access to the commons may result in an increase in the use of pastureland by outsiders. Increase in the invasion of outsiders (Figure 7b) into the pastureland of the Karakul area can also result in the uneven use of the pastureland, though the local community may not allow outsiders to use the pastureland when privatized (Figure 7c). A similar situation is already evident in the Alai Valley, just north of the study area [10,12]. There, the *jailoo* is used both by the villagers of the Alai area and by outsiders coming from far north areas. Most outsiders occupy the *jailoo* near the major roads with good access, and there exists overgrazing in some *jailoo*. Further, the number of livestock in GBAO, including the Karakul area, is rapidly recovering (Figure 2). The uneven use will be further enhanced as the total number of livestock in the Karakul area continues to increase.

Meanwhile, private use of the pastureland (Figure 7c) might be widespread in the Karakul area, as seen elsewhere in the GBAO, because of the law. This scenario will also cause overuse and abandonment/underuse at the same time. Private possession of pastureland decreases livestock mobility. If the pasturelands in the distant places are allocated to poor families, the use of such places becomes difficult. The resultant uneven use of pastureland has been noticed elsewhere in the eastern Tajik Pamirs [16,22,23]. Another study [27] also pointed out that overgrazing around villages has already been seen in the Tajik Pamirs, and many researchers (for example, Reference [34]) pointed out that the privatization of pastureland caused the degradation of pastureland in the Inner Mongolia of China.

Therefore, regardless of privatization or continued use of the commons, uneven use of the pastureland will be developed in the future if they will not introduce the strict management system, which will result in a loss of sustainability. The interviewed local pastoralists told that they would prefer the common use as they currently do. However, free access to pastureland could result in a loss of sustainability as a trade-off if the pastureland is not strictly managed. The use of pastureland without proper management (Figure 7b,c) will ultimately increase not only pasture degradation but poverty among the owners of a small quantity of livestock as well [17].

A shift from the scenarios shown in Figure 7b,c to those shown in Figure 7d,e is important. This shift will cost money because scenarios d and e require the long-distance movement of livestock to the remote pastureland. However, governmental compensation to cover the long-distance movement would be unrealistic. One possible direction might be the introduction of a new goat species with high value, such as cashmere goats. The local pastoralists have had experience in introducing new sheep species in the past, suggesting that introducing new goat species would not be too much of a challenge for them. Then, establishing a marketing system to export cashmere wool will be important for success. Some sheep wool is sold in Sary-Mogol, Kyrgyzstan, which is then sent to Uzbekistan. Because of this, they would be able to clear the hurdle of the marketing system establishment to export the cashmere wool through Sary-Mogol. 

**Figure 9:**
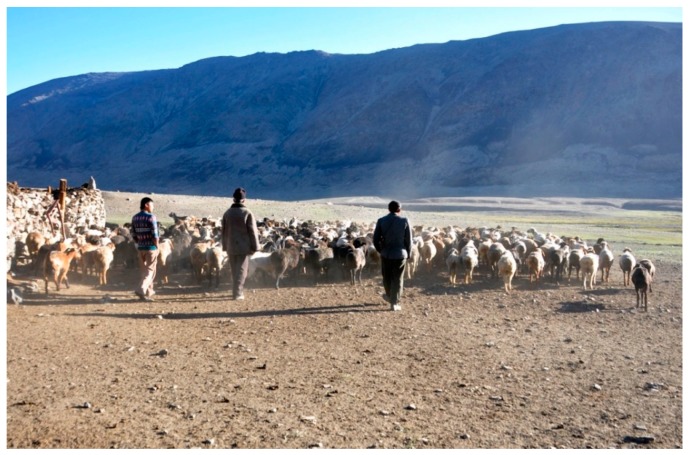
A flock of sheep/goats marching from one of the *jailoo* in the Karakul area to Sary-Mogol for illicit sale (Photograph: T.W., August 2010).

However, blocking access to the local market, which was created by the central government, is another hurdle. No brokers come to the *jailoo* to buy sheep/goats for meat, though some come to the *jailoo* to buy yaks and cows in summer. The Tajik government prohibited the export of livestock from Tajikistan to Kyrgyzstan in 2009 as mentioned before. Therefore, when the local people were interviewed, they would pretend that they shipped their livestock to the market of Murghab. However, de facto, the livestock owners of Karakul ship their sheep/goats to the market of Sary-Mogol in August (Figure 9). Five or six families make a group of 200 to 300 sheep/goats. They can sell a goat for about 4000 to 5000 Som at the market in Sary-Mogol, which is higher than the price in Murghab. Approximately 80% of the sheep/goats in the Karakul area are sold in Sary-Mogol. This demonstrates the importance of the accessibility to Sary-Mogol for the people of Karakul. The law enforced in 2009 to prohibit the export of livestock (sheep/goats) to Sary-Mogol has led to a clandestine/illicit local economy. The traditional sales to Sary-Mogol by Karakul pastoralists are vital not only to maintaining the sustainability of their economy, but also to augment the sustainability of the pastureland use for the future.

Pastureland management of Kyrgyzstan [35] may be a model for the Tajik Pamirs. The Kyrgyz government made a National Land Code in 2002 and a law for pastureland in 2009, which was enforced in 2010. Pastureland is regarded as the residents’ joint stewardship, namely as the commons. A local community’s head assigns some of the residents to make *Jaiyt Komitet,* a local pasture committee, in every settlement to manage their pastureland [11,12]. However, the committee members are often selected because they are relatives or close friends of the community’s head, not because they are capable of management. The selection method of the committee members is important to the function of the pasture committee. As the level of governance by the pasture committee needs to be empowered in southern Kyrgyzstan [13], a similar management committee needs to be developed in the study area which possesses empowerment; currently, the village authority with the help from the pastoralists are supposed to carry out the role as mentioned earlier. The Tajik government recognizes the importance of establishing a pasture-user association [18]. Theoretically with a functional pasture committee, even privatized pastureland would be used evenly (Figure 7e), although the reality in the future might be different. Developing a new management system with a functional pasture committee is key to succeeding with future sustainability.

### 4.3. Limitations

This study clarified the locations of *jailoo* and the patterns and timing of the seasonal pastoral movement. However, we still need detailed information on *kyshtoo* (winter pasture). As mentioned earlier, many *kyshtoo* are located in the buffer zone (Figure 3), to which only the local pastoralists are allowed access to [36]. Furthermore, visiting the Karakul area in winter is a challenge. Understanding the pastoral practice in winter is an important research agenda for future sustainability because the shortage of winter fodder is a limiting factor for the number of livestock in eastern GBAO [37,38].

## 5. Conclusions

After pastoralism was transformed following independence in 1991 to management by personal farmers (*fermer*), livestock owners have continued pastoralism using the altitudinal difference in the Karakul area of the north-eastern Tajik Pamirs. Four groups that use different *jailoo* in summer were identified in the study area: (1) the Kyzyl-Art Valley, (2) the Akjilga Valley, (3) the Kokuibel Valley, and (4) the Muzkol Valley. In 2010, the total number of *jailoo* used in the area was 28, and in 2012 it was 23. Livestock was moved from the *jailoo* to the *kyshtoo* around the village in winter. Each group pastures their livestock every day, a system that is called *novad*. In addition to *jailoo* and *kyshtoo*, they also practice their pastoralism on the *küzdöö* (spring pastureland) and the *bäärlöö* (autumn pastureland), although these two pasture types appear to be less important because of the short period of stay there.

Pastureland in the Karakul area has not yet been privatized, unlike some other areas in the eastern Tajik Pamirs. Pastureland is the target of ‘modernization projects’ in the countries of the Pamirs [1]. The Karakul area continues the use of traditional pastoral practices and has not yet been affected by such projects or by the law enabling privatization of pastureland. Nevertheless, there are some signs of the uneven use of pastureland. The local pastoralists highlighted that they would prefer to continue with the common use of pastureland; however, there will be trade-offs of free access to the pastureland unless a local pasture committee with strict management is established. Further monitoring of the transformation of pastureland use is necessary to understand how the sustainability of this area will change in the future. 

## Figures and Tables

**Table 1:** The summary of the measured climatological data in Sai-Konush Jailoo, Jalang Jailoo, and the Karakul village from August 2014 to July 2015.

Elements	Sai-Konush (4348 m)	Jalang (4094 m)	Karakul (3920 m)
Precipitation for 7 months (excluding winter)	73.0 mm	39.2 mm	26.2 mm
Annual average air temperature	−2.7	−1.1	−0.1
Average soil moisture from May to August (min–max)	12.1% (7.2–21.3%)	13.8 * (8.7–24.9%)	3.9% (2.8–5.2%)

* Data from 1 May to 23 August only. Sai-Konush Jailoo: Ak5 in Figure 3, Jalang Jailoo: Ko6 in Figure 3.
